# Universal dimensions of visual representation

**DOI:** 10.1126/sciadv.adw7697

**Published:** 2025-07-02

**Authors:** Zirui Chen, Michael F. Bonner

**Affiliations:** Department of Cognitive Science, Johns Hopkins University, Baltimore 21218, USA.

## Abstract

Do visual neural networks learn brain-aligned representations because they share architectural constraints and task objectives with biological vision or because they share universal features of natural image processing? We characterized the universality of hundreds of thousands of representational dimensions from networks with different architectures, tasks, and training data. We found that diverse networks learn to represent natural images using a shared set of latent dimensions, despite having highly distinct designs. Next, by comparing these networks with human brain representations measured with functional magnetic resonance imaging, we found that the most brain-aligned representations in neural networks are those that are universal and independent of a network’s specific characteristics. Each network can be reduced to fewer than 10 of its most universal dimensions with little impact on its representational similarity to the brain. These results suggest that the underlying similarities between artificial and biological vision are primarily governed by a core set of universal representations that are convergently learned by diverse systems.

## Teaser

Probing neural representations reveals universal aspects of vision in artificial and biological networks.

## INTRODUCTION

Deep neural networks (DNNs) have the ability to simulate the representations of biological vision (*1*–*4*). However, because of their immense complexity, the principles that govern the brain-aligned representations of DNNs remain poorly understood.

A leading approach interprets neural network representations in terms of their architectures and task objectives, which are thought to function as key constraints on a network’s learned representations (*5*–*9*). However, an alternative possibility is that the brain-aligned representations of neural networks are not contingent on specific optimization constraints but instead reflect universal aspects of natural image representation that emerge in diverse systems (*4*, *10*, *11*).

Here, we sought to determine whether the representations that neural networks share with human vision are universal across networks. We examined more than 200,000 dimensions of natural image representation in DNNs with varied designs. Our analyses revealed the existence of universal dimensions that are shared across networks and emerge under highly varied optimization conditions. Universal dimensions were observed across the full depth of network layers and across a variety of architectures and task objectives. Visualizations of these dimensions show that they encode not only low-level image statistics but also higher-level semantic properties. We next compared these dimensions to the representations of the human brain measured with functional magnetic resonance imaging (fMRI), and we found that universal dimensions are highly brain aligned and underlie conventional measures of representational similarity between neural networks and the visual cortex. Together, these findings demonstrate the notable degree to which the shared properties of artificial and biological vision correspond to general-purpose representations that have little to do with the details of a network’s architecture or task objective.

## RESULTS

### Assessing universality and brain similarity

We sought to compare two fundamental quantities of representational dimensions in neural networks: (i) their universality across varied networks and (ii) their similarity to human brain representations. Here, we briefly describe how we computed these two quantities. A more detailed description is provided in Materials and Methods.

**Fig. 1.**
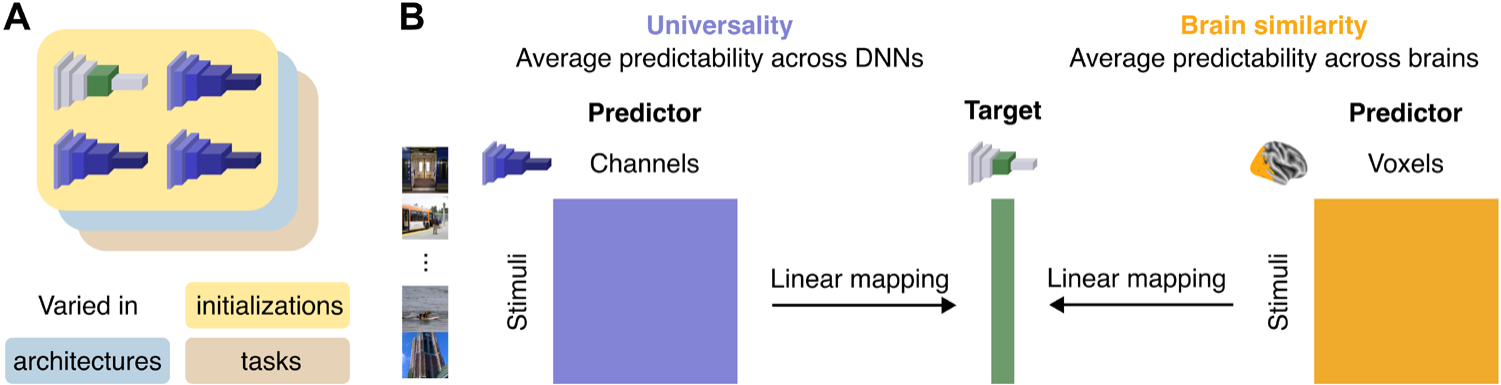
Overview of a method for computing universality and brain similarity of network dimensions. (**A**) Four sets of DNNs were analyzed, including three sets of trained models that varied in their random initializations, architectures, or task objectives and one set of untrained models with different initializations. (**B**) Universality and brain similarity were defined as the average prediction accuracy of a latent dimension from a target network when using the activations of other networks or the fMRI activations of the human brain as predictors. Dimensions that can be consistently predicted from the representations of other networks have high universality. Dimensions that can be consistently predicted from the representations of the human brain have high brain similarity.

As illustrated in Fig. 1, we characterized universality and brain similarity by examining the activations of networks and the human brain to a subset of natural images from the Microsoft Common Objects in Context (COCO) image database (*12*). For each latent dimension *d* of a network’s activations (i.e., each principal component), we computed a universality metric by obtaining its median predictability from the activations of *m* other networks

$${\text{Universality}}_{d}=\text{median}\left(r_{d,1},r_{d,2},\ldots ,r_{d,m}\right)\;$$ (1)

where $r_{d,m}$ is the prediction accuracy of a cross-validated linear regression with dimension *d* as the predictand and network *m* as the predictor. We performed these analyses on the principal components (PCs) of a network’s activations so that each dimension *d* is sampled from an orthogonal basis rather than from a set of potentially redundant neurons. The motivation for this metric is to quantify the average degree to which a dimension in one network is shared with the representations of other networks. Dimensions that are shared across all networks will have universality scores close to 1, while dimensions that only emerge under specific network configurations will have universality scores close to 0.

We examined the universality of representational dimensions among several sets of vision networks that varied in their random initializations, architectures, or task objectives. The specific networks used for these analyses are described in Materials and Methods and listed in tables S1 and S2. We sought to focus on the kinds of features represented by different networks rather than their spatial dimensions. Therefore, we applied global max pooling to remove spatial information from the activations of all network layers. We then extracted all orthogonal dimensions from each network layer through principal components analysis (PCA). PCA was applied to each layer’s pooled activations from 72,128 COCO images, and all PCs were retained. We then transformed 872 COCO images to the PC basis for each layer and computed universality and brain similarity metrics. We focused on layerwise PCs so that we could examine trends in universality and brain similarity across the depth of each network.

To compute universality scores, we used cross-validated ridge regression. For a given dimension in a target network, we sought to predict its activations as a linear combination of the activations from another predictor network, and we repeated this procedure using all other networks as predictors. For these regressions, we concatenated the layers of the predictor network (as opposed to performing separate regressions for each layer) so that we could obtain a single prediction score for each distinct predictor network. We then computed the mean Pearson correlation between the predicted and actual responses of the target dimension across all cross-validation folds, and we repeated this process, using every network other than the target as the predictor. We obtained a universality score by taking the median correlation across all predictor networks. We used median instead of mean to ensure that the final summary statistic was not driven by a small subset of predictor networks with exceptionally high or low scores.

For each dimension *d* of a network’s activations, we also computed a brain similarity metric by obtaining its mean predictability from the fMRI activations of *n* human brains

$$\text{Brain}\;{\text{similarity}}_{d}=\text{mean}\left(r_{d,1},r_{d,2},\ldots ,r_{d,n}\right)\;$$ (2)

where $r_{d,n}$ is the prediction accuracy of a cross-validated linear regression with dimension *d* as the predictand and subject *n* as the predictor. Here, the motivation is to quantify the average degree to which a network dimension is shared with the representations of the human visual cortex. Dimensions that are shared between networks and humans will have brain similarity scores close to 1, while dimensions that are not shared with humans will have brain similarity scores close to 0.

To assess brain similarity, we compared network representations with image-evoked fMRI responses from the Natural Scenes Dataset (NSD) (*13*), which is the largest existing fMRI dataset of natural scene perception in the human brain. We focused on a portion of this dataset that contains fMRI responses to 872 images shown to each of eight participants. This dataset is ideally suited for assessing whether the dimensions of natural image representations in neural networks can also be found in the representations of the human brain. For our main analyses, we focused on a general region of interest (ROI) that included all voxels in the visual cortex whose activity was modulated by the presentation of visual stimuli (Fig. 1).

To compute brain similarity scores, we again used cross-validated ridge regression. Given a target dimension from a network, we sought to predict its activations as a linear combination of the fMRI activations from one subject. We computed the mean Pearson correlation between the predicted and actual responses of the target dimension across all cross-validation folds, and we repeated this procedure for all fMRI subjects. We then computed a brain similarity score by taking the mean correlation across all subjects.

### Universality across initialization weights

We used universality and brain similarity to address a central question: Are there universal dimensions of natural image representation that are shared by neural networks and humans? We first performed these analyses for a set of networks that were initialized with different random weights but were otherwise identical (i.e., same architecture, task, and training data)—a setting in which we naturally expect to find some degree of shared network dimensions. Specifically, these networks were 20 ResNet-18 architectures trained on image classification using the Tiny ImageNet dataset (*14*–*16*). For each dimension in each network layer, we computed its universality using the other networks as predictors. We examined network layers spanning the full range of model depth, except for the final classification layer. We iterated this analysis over a total of 36,596 dimensions across all networks.

**Fig. 2.**
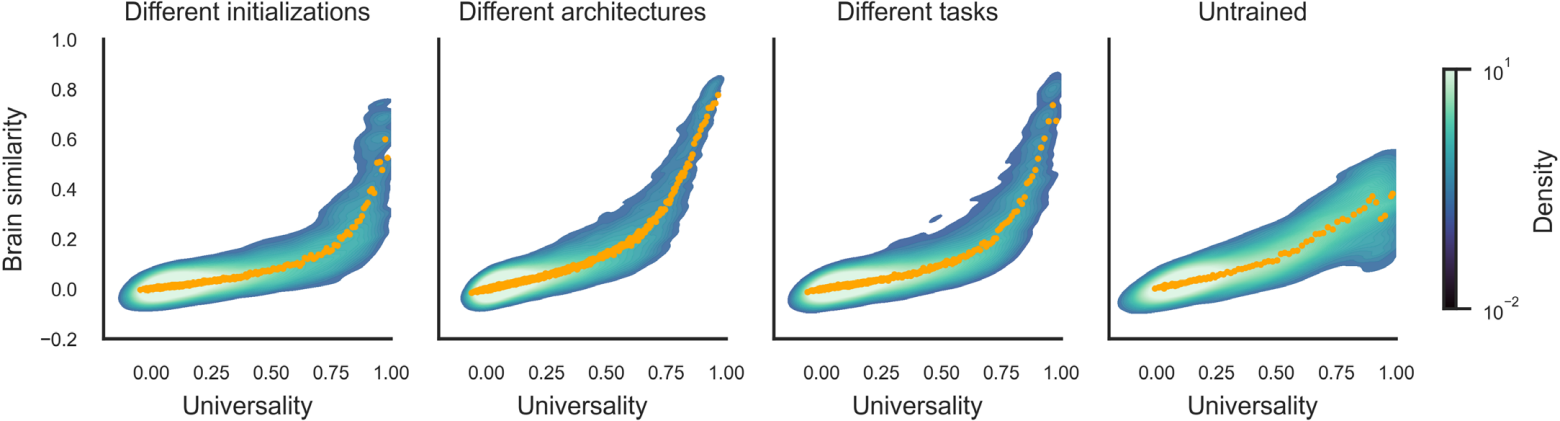
Universality and brain similarity of network dimensions. Universality and brain similarity were computed for representational dimensions in four sets of DNNs. These included three sets of trained networks with varied initializations, architectures, and task objectives and one set of untrained networks. These metrics were computed for the PCs of network activations extracted from the sampled layers of each network. Universality scores reflect the degree to which a representational dimension is shared across all networks in a set, and brain similarity scores reflect the degree to which a representational dimension is shared with the human visual system. Measurements of the human visual cortex activity were obtained from the Natural Scenes fMRI Dataset using a general ROI that included all visually responsive voxels (*13*). Universality and brain similarity scores are plotted for all analyzed network dimensions. These plots show the density of dimensions on a logarithmic scale, with densities computed using kernel density estimation. The orange dots show the mean universality and brain similarity scores for equally sized quantiles of 100 dimensions along the *x* axis. These plots show similar trends for all three sets of trained models (the first three plots on the left). Specifically, they exhibit a high density of points near the origin, showing that most dimensions are idiosyncratic to each network and not shared with the human brain. However, they also contain a subset of dimensions with exceptionally high universality and brain similarity scores. These latter dimensions correspond to representations that are consistently learned by all networks within a set and also strongly shared with the visual representations of the human brain. In contrast, untrained networks (right) can also have shared dimensions, but these shared untrained dimensions have relatively weak brain similarity scores.

As shown in the left panel of Fig. 2, we observed universality scores spanning the full range from 0 to 1, with a high density of points around 0, indicating that most dimensions are idiosyncratic. The high density of idiosyncratic dimensions could reflect representations present at initialization that remain largely unchanged during training, or they could reflect unique representational strategies learned by specific network instances. In contrast, the universal dimensions at the other end of the scale reflect convergent representations that reliably emerge in all networks despite differences in their starting points. Notably, these universal dimensions account for a relatively small subset of the total number of network dimensions, as illustrated by the lower density of points at the high end of the universality axis.

We next compared these network dimensions to human brain representations, and we found that the universal dimensions exhibit exceptionally strong brain similarity scores (Fig. 2). Universality and brain similarity are strongly correlated (assessed with a nonparametric Spearman correlation, ρ = 0.61, and permutation test, *P* < 0.0001). This demonstrates that among the many network dimensions examined here, only those that are invariably learned by networks with different initial conditions are also strongly shared with the representations of the human visual system. We wondered whether this effect could be fully explained by the ranks of the network PCs, as the most universal dimensions are expected to be high-variance components. To address this, we performed a partial correlation analysis controlling for PC rank. This analysis shows that the correlation is reduced but remains significant (fig. S1; Spearman’s ρ = 0.29, and permutation test, *P* < 0.0001). Thus, while PC ranks are relevant to the relationship between universality and brain similarity, this relationship cannot be reduced to an effect of PC rank.

**Fig. 3.**
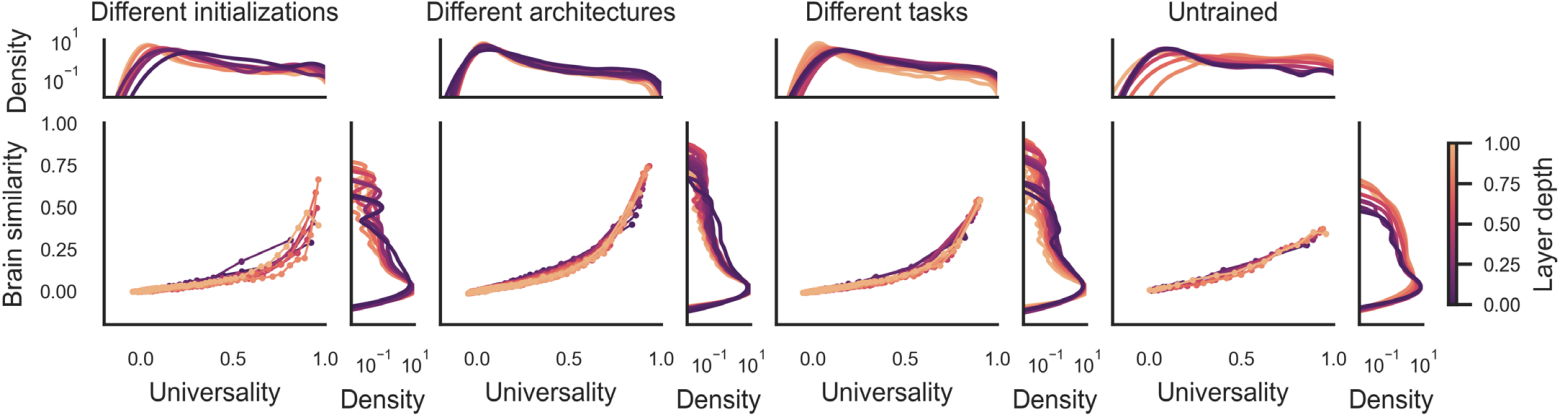
Universality and brain similarity across network layers. These plots show the universality and brain similarity scores for individual network layers spanning the full depth of each network. Four sets of DNNs were examined, including three sets of trained networks with varied initializations, architectures, and tasks and one set of untrained networks. The analyses are the same as in Fig. 2, but here, the results are plotted as the average values for individual layers, which are labeled according to their relative depth. Further details of these network layers are included in the Supplementary Materials (*46*). Average universality and brain similarity scores were computed for equally sized quantiles of 100 dimensions along the *x* axis for each layer. Panels on the sides of each plot show the density of dimensions on a logarithmic scale computed using kernel density estimation. As in Fig. 2, these plots exhibit a high density of points near the origin, which means that across all sampled layers, most dimensions are idiosyncratic and not shared with the human brain. However, the three sets of trained networks (the first three plots on the left) also contain a subset of dimensions at the right end of each plot that has exceptionally high universality and brain similarity scores. These layerwise plots show that a consistent trend is observed across all sampled layers and that universal dimensions are not restricted to early network layers.

In follow-up analyses, we found that the trend in Fig. 2 was consistently observed in each network layer (Fig. 3), each individual network (fig. S2), each individual fMRI subject (fig. S3), and in multiple ROIs in the visual cortex (fig. S4). The observation of this effect in all network layers shows that universality is not restricted to low-level features in early layers but instead extends across the full depth of the network. We emphasize that the universality and brain similarity metrics in our analysis pipeline are not guaranteed to be related to one another. Using simulated data, we can trivially obtain a range of universality and brain similarity scores while observing no positive relationship between these two metrics (fig. S5). This simulation shows that our method could detect a divergence between universality and brain similarity if such a divergence were actually present in the data. Furthermore, we recomputed the universality and brain similarity metrics using other mapping procedures to ensure that our findings are not contingent on the specific regression method that we used. These analyses showed that the variation in universality and brain similarity across network dimensions is highly consistent when using unregularized regression instead of ridge regression and even when using a one-to-one mapping procedure without any regression-based reweighting (fig. S6). Thus, the observed trends are highly robust and not contingent on regularization or regression-based mapping in general. Together, these findings show that among a preponderance of idiosyncratic network dimensions, there exists a smaller subset of highly convergent dimensions that is learned by different network instances and shared with human vision.

### Universality across architectures and tasks

We next sought to determine whether universal dimensions can be detected among networks with varied architectures and tasks. To this end, we quantified universality and brain similarity for two sets of models. The first was a set of models with different architectures but trained on the same task. Specifically, we examined 19 networks trained to perform ImageNet classification using varied architectures, including convolutional models, vision transformers, and MLP-Mixers. The second was a set of nine models with the same architecture (ResNet-50) but trained on different tasks. The tasks included object classification and a variety of self-supervised tasks, such as contrastive learning, identifying image rotations, and solving jigsaw puzzles. For all models, we examined layers spanning the full network depth, except for the final layer. Further details about the models can be found in tables S1 and S2. In total, we examined 149,743 dimensions in the set of varied architectures and 43,132 dimensions in the set of varied tasks.

The findings for these two sets of models were unexpectedly consistent with those observed for models with varied initializations (Fig. 2). We again found that most dimensions are idiosyncratic (i.e., specific to a model) and not shared with the brain, as shown by the high density of points near the origin. Again, we also found that a subset of dimensions exhibits exceptionally high scores on both the universality and brain similarity metrics. These latter dimensions correspond to representations that reliably emerge across many models despite variations in their architectures and the tasks that they were trained to perform. Furthermore, the generality of these representations extends beyond artificial vision, as they are also strongly shared with the representations of the human visual system. These findings are highly similar when considering networks that vary in either architectures or task objectives. This suggests that the underlying similarities among the representations of these networks—as well as their similarities to human vision—largely reflect highly general properties of image representations in DNNs.

We also note that there is a paucity of points in the upper left quadrant of the plots in Fig. 2. Points in this quadrant would correspond to representations shared with the brain but learned only by networks with specific optimization constraints—namely, specific architectures or tasks. The lack of points in this quadrant suggests that the details of architectures and tasks have a relatively minor role in shaping the brain-aligned representations of neural networks.

In follow-up analyses, we again found that universality and brain similarity were strongly correlated (Spearman’s ρ = 0.65 for the different-architecture set and ρ = 0.68 for the different-task set, both *P* < 0.0001). Again, the partial correlations were significant when controlling for PC rank (Spearman’s ρ = 0.40 for the different-architecture set and ρ = 0.32 for the different-task set, both *P* < 0.0001). We also found that these results are highly robust—they were observed in each network layer (Fig. 3), each individual network (fig. S2), each individual fMRI subject (fig. S3), and in multiple ROIs in the visual cortex (fig. S4). Together, these findings reveal the degree to which vision systems with varied architectures and task objectives can nonetheless converge on a set of general-purpose representations that are shared not only across models but also between artificial and biological vision.

### Universality across untrained networks

Our analyses thus far have focused on sets of trained neural networks, and we interpreted the dimensions in the upper right quadrant of the plots in Fig. 2 as learned representations. However, we expect that shared dimensions can also be found among sets of untrained models due to statistical regularities in the activations that natural images elicit in networks with random filters. We thus wondered whether our findings for the trained networks could be explained by the statistics of image activations alone—without any need for learning—or whether they diverge from the trends observed in randomly initialized networks. To address this question, we examined 20 ResNet-18 architectures that were randomly initialized with the same seeds as the trained models presented in the left panel of Fig. 2. We followed the same procedures as in the preceding analyses. For each dimension in each network layer, we computed its universality using the other networks as predictors, and we iterated this analysis over a total of 9413 dimensions from all networks.

These analyses showed that, as expected, there is a wide range of universality scores for the untrained network dimensions, with some dimensions that are found in all networks (Fig. 2). These universal dimensions of untrained networks correspond to representations that consistently emerge when propagating natural images through a hierarchy of random convolutional filters. They are thus due to image statistics alone and not learned representational properties. However, the relationship between universality and brain similarity diverges from the relationship that was observed for trained models. Specifically, for untrained networks, we observe a shallow and approximately linear relationship between universality and brain similarity, whereas, for trained networks, brain similarity exhibits a sharp nonlinear increase at the high end of the universality axis. As a result, the shared dimensions of untrained networks have substantially lower brain similarity scores than the shared dimensions of trained networks. As in the previous sets of analyses, we again found that universality and brain similarity were strongly correlated (Spearman’s ρ = 0.84, *P* < 0.0001), and partial correlation was significant when controlling for PC rank (Spearman’s ρ = 0.50, *P* < 0.0001). We also found that these results were consistent in each network layer (Fig. 3), each individual network (fig. S2), each individual fMRI subject (fig. S3), and multiple ROIs in the visual cortex (fig. S4). In sum, when comparing the trends for trained and untrained networks, the findings demonstrate that the universal dimensions of trained networks reflect learned representational properties that cannot be explained by image statistics and random features alone.

Last, we performed an analysis to determine whether the observed trends in brain similarity might be affected by the reliability of the fMRI data. Specifically, we wondered whether low brain similarity scores might reflect low reliability in the fMRI data rather than a mismatch between a network and the brain. The brain similarity scores in our analyses can only be as high as the reliability of the underlying fMRI data across subjects. Hence, it is possible to compute a straightforward metric of between-subject reliability and adjust the brain similarity scores by this metric. As shown in fig. S7, the observed trends in brain similarity for all four sets of models are qualitatively unchanged after adjusting for reliability.

### Universality and the visual hierarchy

Previous work has shown that in many neural networks trained on natural images, the first layer contains general-purpose V1-like filters tuned to orientation, frequency, and color, whereas subsequent layers contain filters that appear to be increasingly specialized (*17*, *18*). This suggests the possibility that universality may only be prominent in early network layers and then rapidly diminish across the network hierarchy. To address this possibility, we examined the universality and brain similarity of network representations in individual layers along the full depth of each network. Figure 3 shows the results of these analyses for all sets of models. Across all sets of trained models, we did not find a strong layer effect but instead relatively similar distributions of universality scores at all sampled layers, with highly universal dimensions detected even in the deepest layers that we examined. Furthermore, these analyses show that the relationship between universality and brain similarity is consistent across layers, and we found that in all layers, they were strongly correlated (mean Spearman’s ρ = 0.70, SD = 0.09, all *P* < 0.0001) and partial correlations were significant when controlling for PC rank (mean Spearman’s ρ = 0.37, SD = 0.09, all *P* < 0.0001). Thus, these findings suggest that at all levels of network depth, we can find general-purpose representations that are reliably learned by diverse networks and strongly shared with the human brain.

### Universal dimensions and high-level image properties

The findings from the previous section show that universal dimensions are not restricted to early network layers. However, these findings do not directly address the question of whether the shared dimensions of later layers represent high-level semantic properties or low-level image statistics, such as luminance gradients and spatial frequency distributions. To address this question, we performed exploratory visualization analyses of the universal dimensions in later network layers. Specifically, we examined the penultimate layer of the models from the varied-task set, as shown in the middle right panel of Fig. 2. We focused on the varied-task set because the models in this set have the same ResNet-50 architecture, which allowed us to examine representations from the same targeted layer in all networks. We concatenated the dimensions from the penultimate layer across all networks and ranked their universality scores. We then selected the top 100 dimensions with the highest universality scores and visualized the image representations of these 100 dimensions in a two-dimensional (2D) space using uniform manifold approximation and projection (UMAP) (*19*).

**Fig. 4.**
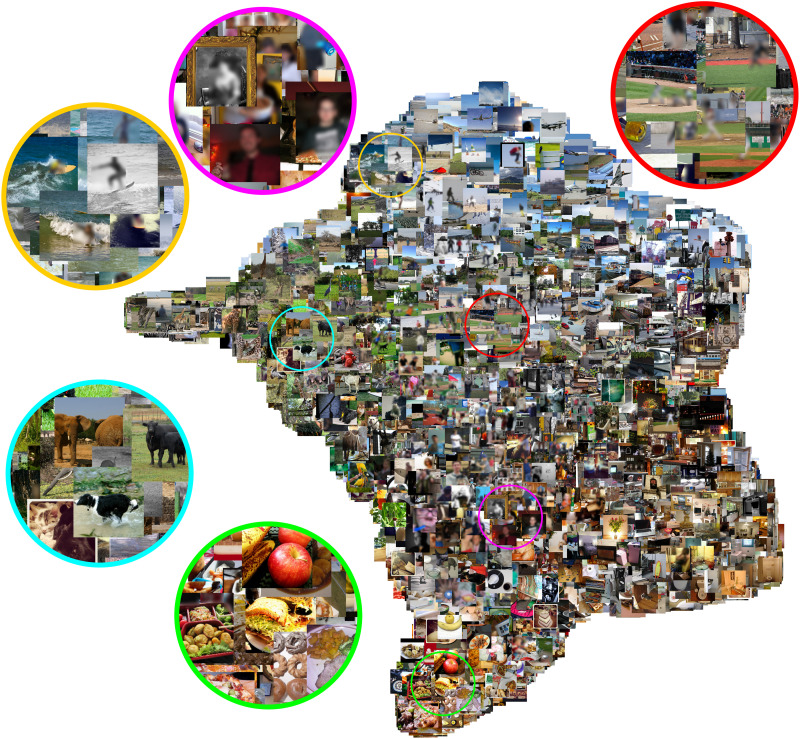
2D visualization of high-level universal representations. Image activations for the 100 most universal dimensions from a high-level network layer were embedded in two dimensions using UMAP. Specifically, image activations were obtained for the top 100 dimensions with the highest universality scores in the penultimate layer from the set of models trained on different tasks. For visualization purposes, this figure only includes images shown to a single subject. This plot shows that universal dimensions do not reflect low-level image features but instead capture high-level properties that group images into semantically related clusters, some of which are highlighted here, including animals, food, sports, and people. In contrast, fig. S8 shows a visualization of the 100 least universal dimensions from the same network layer, and it shows no clear semantic organization.

As shown in Fig. 4, these representations exhibit rich high-level organization, with images grouped into clusters of semantically related items, such as people, sports, animals, and food. To verify our interpretation of this visualization, we used representational similarity analysis (RSA) to compare the 100 selected model dimensions of all 73,000 images from the NSD dataset with semantic embeddings based on image captions. The semantic embeddings were obtained from a sentence transformer and averaged across all five human-annotated captions (*20*, *21*). Representational dissimilarity matrices (RDMs) for the semantic embeddings and the network dimensions were computed using Pearson distance. This analysis revealed substantial representational similarity between the semantic embeddings and the high-universality model dimensions (Spearman’s ρ = 0.37). For comparison, we also generated UMAP embeddings of the 100 dimensions with the lowest universality scores from the same ResNet-50 layer. We found that these idiosyncratic dimensions exhibit no clear semantic organization (fig. S8) and no correlation with the semantic embeddings (ρ = 0.00). Thus, the semantic organization seen in Fig. 4 is not an artifact of the UMAP procedure itself; rather, it is an inherent property of the universal representations. We also generated UMAP embeddings of the top 100 universal dimensions in the penultimate layer of the untrained-network set, which is shown in the rightmost panel of Fig. 2. In contrast to the trained networks, the universal dimensions of untrained networks emphasize prominent low-level features, such as coarse luminance gradients (fig. S9), which again are not correlated with the semantic embeddings (ρ = 0.05). Together, these visualization analyses show that the universal representations of high-level layers in trained networks encode high-level image properties that group images into semantically meaningful clusters. This suggests that there are common organizing principles of high-level image semantics that are universally learned.

### Universal dimensions and RSA

**Fig. 5.**
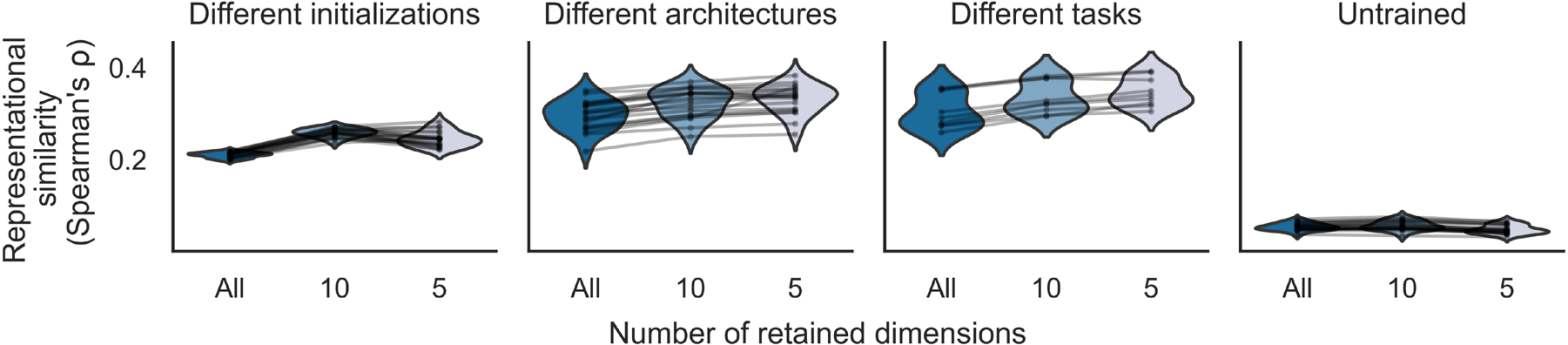
Universal dimensions underlie the results of conventional RSAs. RSA was used to compare the representations of neural networks and the visual cortex. These analyses were performed using the same general ROI in the visual cortex and the same sets of neural networks as in Figs. 2 and 3. RDMs were created by calculating Pearson correlation distances for pairwise comparisons of image representations within each network and each fMRI subject. Representational similarities were obtained by calculating the Spearman correlation between the RDMs for a network and an fMRI subject. These representational similarities were averaged across subjects. For each network, the best-performing layer was selected using a set of training data, and the final representational similarity was computed on the held-out test data. These analyses show the results of RSA for networks whose representations were either intact or reduced to subspaces of their top 10 or 5 universal dimensions. In these plots, each dot is a network, and lines connect different versions of a network containing all, 10, or 5 dimensions. The violin plots show distributions of representational similarities across networks. Even after markedly reducing the networks to just 10 or 5 universal dimensions, the representational similarities exhibit little or no decrease—rather, for all three sets of trained networks, the representational similarities slightly improve. These results demonstrate that conventional measures of representational similarity between neural networks and the visual cortex are largely driven by the subspaces of universal dimensions contained within each network. Similar trends were observed within each individual subject and in other ROIs, as shown in figs. S10 and S11.

Our findings thus far show that the universal dimensions of networks can be strongly predicted from human brain representations. We next sought to evaluate the effect of universal dimensions on a conventional RSA (*22*). As shown in fig. S12, universal dimensions tend to be low-rank PCs, which suggests that they are important to the representational geometry of each network. To test this prediction, we performed a targeted analysis to determine whether universal dimensions drive the representational similarity scores obtained from comparisons of neural networks with the visual cortex. To do so, we conducted a standard RSA on networks with representations reduced to low-dimensional subspaces of their most universal dimensions. We performed this analysis for all sets of networks examined in Fig. 2. Following the RSA procedures in (*3*), we split the stimuli into training and test sets. We selected the best-performing layer from each network on the training set and computed the representational similarity for the selected layers on the test set. We then reduced each network to a subset of its most universal dimensions and computed the representational similarity for these reduced model representations. We analyzed the same general ROI in the visual cortex as in the preceding analyses. We found that even when the networks are reduced to just 10 or 5 universal dimensions, their representational similarities exhibit little or no decrease—rather, for all three sets of trained networks, they slightly improve (Fig. 5). Similar results were observed when performing these analyses in individual subjects (fig. S10) and other ROIs (fig. S11). These findings suggest that the representational similarities between neural networks and the visual cortex are largely driven by subspaces of network dimensions that are universal.

## DISCUSSION

Our work reveals universal dimensions of natural image representation that are learned by artificial vision systems and shared with the human brain. These dimensions emerge in diverse neural networks despite variation in their architectures and task objectives. The role of these dimensions in vision appears to be general-purpose—they are not specialized for any single task but instead support many downstream objectives. Universal representations are found at all levels of visual processing in DNNs, from low-level image properties in early layers to high-level semantics in late layers. Together, these findings suggest that machines and humans share a set of general-purpose visual representations that can be learned under highly varied conditions.

Deep learning is now the standard framework for computational modeling in neuroscience, and many previous efforts have sought to understand these deep learning models in terms of their specialization: that is, what objectives they are specialized for and what specific network characteristics underlie their similarities to the brain (*5*–*9*). Our work views the representations of DNNs from a different perspective. Rather than searching for specific model characteristics that might be associated with stronger alignment with the brain, we sought to find the elements of network representations that are instead invariant across models. Using this approach, we found that crucial aspects of DNN representations—those that are most strongly shared with the human brain—are, to a degree, independent of the network characteristics that many previous studies have emphasized. The invariance of these representations implies that they are not primarily governed by the details of a network’s architecture or task objective but instead by more general principles of natural image representation in deep vision systems (*10*, *11*).

We want to emphasize that our results do not indicate a complete lack of meaningful representational differences across networks. Our work reveals that important aspects of network representations are universal, but this does not imply that all networks will learn these universal representations to the same extent or with the same level of quality or that all relevant aspects of a network’s representations will be universal. We can see in Fig. 2 that many of the dimensions in the networks studied here have intermediate levels of both universality and brain similarity. These dimensions partly differ across networks while nonetheless exhibiting a nonnegligible degree of brain alignment. Furthermore, when considering the RSA results in Fig. 5, we can see that there is substantial variability across the networks within each set. Thus, although these networks share universal dimensions, this does not mean that their representational similarity to the visual cortex will be identical. These examples illustrate the point that despite the existence of universal dimensions, there may still be meaningful differences across networks. Much previous work has shown that differences in architecture, task objective, and training data can influence representational similarity to the visual cortex [e.g., (*23*–*25*)]. Thus, our claim is not that researchers should abandon efforts to identify specific aspects of network design that lead to better representational models of the brain. Instead, our claim is that there exists a notably universal set of natural image representations that is worth understanding in its own right and that requires us to focus not on what differs across networks but rather on what is shared.

In our analyses, we focused on the PCs of each network’s representations. We chose to focus on PCs for two reasons. First, this approach allowed us to specifically characterize the proportion of distinct (i.e., orthogonal) dimensions that have high universality, which we found to be small relative to the hundreds of thousands of dimensions we analyzed. Second, many previous studies comparing representations across networks have focused on principal axes of variance, and both empirical and theoretical works suggest that the information a network learns is primarily reflected in its lower-rank PCs (*4*, *26*, *27*). However, there are alternative dimensions that could be explored, including nonorthogonal bases, sparse bases, or nonlinear manifold coordinates (*28*–*30*). An intriguing question for future work is whether alternative coordinate systems could reveal an even greater degree of universality across networks.

Universality and PC rank are fundamentally distinct attributes of representational dimensions, and one should not assume that all low-rank PCs are universal. For example, variations in tasks, architectures, and training procedures can markedly alter the shape of the representational eigenspectrum and, thus, the interpretation of PC ranks (*4*, *31*, *32*), and it appears that some modern architectures and self-supervised learning objectives can yield low-rank PCs that are highly idiosyncratic (*33*). To illustrate this in our own data, we plotted the distribution of universality scores for three different decades of PC ranks (fig. S12). As expected, we find that low-rank PCs typically have high universality scores, but, importantly, within each decade of PC ranks, the universality scores span a wide range. Universality scores for the top 10 PCs can range as low as those in the third decade of PC ranks. This means that while universal dimensions tend to be low rank, not all low-rank PCs are universal. Nonetheless, an important goal for future work is to gain a deeper understanding of the underlying mechanisms that govern the variance and universality of network dimensions and the relationship between them.

The universal dimensions that we examined in this work emerge in models that are trained with a shared visual diet, despite differences in architecture and task objective. An important direction for future work is to understand whether models trained with different sets of images also learn shared representations. More broadly, it may be possible to quantify how the overlap of training data distributions governs the degree to which networks learned shared representations (*34*). As a first step in this direction, we performed a preliminary analysis of the shared dimensions across a set of publicly available networks matched on architecture and task objective but trained on distinct sets of natural images (including object-centric images, complex scenes with multiple objects, faces, and large-scale places) (*35*). The findings suggest that even when networks are trained on highly distinct sets of natural images, they nonetheless learn a subset of strongly shared representational dimensions, and as in our other analyses, it is these shared dimensions that are most similar to the representations of the human visual system (fig. S13). This preliminary analysis suggests that the universal dimensions examined here reflect highly general properties of natural images, but more work is needed to precisely determine the relationship between universality and the statistics of training set distributions.

The universality metric computed in our work is always defined relative to some set of networks. It is important to keep this in mind when interpreting universality scores. Specifically, while this metric can be used to identify which representational features are shared across a given set of networks, it should not be interpreted as identifying features that will invariably be shared with any arbitrary network or with the brain. We could trivially demonstrate this point by training a set of networks to learn the same useless feature—for instance, the number of red pixels in an image. This feature would have a high universality score for this set of networks, but it would have little in common with conventional DNNs or with the brain. We can also see an example of this point when considering the untrained networks in Fig. 2. Untrained networks have dimensions with high universality scores (i.e., dimensions that are shared with other untrained networks). However, relative to the universal dimensions of trained networks, these untrained network dimensions have much lower brain similarity scores. Hence, universality means something different when considering untrained versus trained networks, and more generally, universality scores need to be interpreted in the context of the model set being evaluated. An important limitation of our work is that we have thus far evaluated universality across conventional neural networks developed by the computer vision community and trained on natural images. There are thus important open questions about how widely the universal dimensions examined here will generalize when considering even more diverse architectures and objective functions or when considering other domains of visual images, such as drawings.

Our findings suggest several other exciting directions for future work. First, our approach could be extended beyond vision models to examine the representational dimensions that are shared across vision and language. Previous work has shown that language-model embeddings of object names and scene captions are predictive of image representations in the high-level visual cortex (*24**,* *36**,* *37*). An open question is whether networks trained on language data alone learn the same universal dimensions of natural scene representation as image-trained networks. Second, our findings show that universal dimensions emerge in networks despite differences in the tasks that the networks are optimized to perform. This raises the intriguing possibility that universal dimensions could be hard coded into networks at initialization, potentially making the learning process faster and more data efficient. Third, in this work, our goal was to characterize the universality of each network dimension, but future work could take a different approach by trying to align multiple networks along a common set of axes to identify a core set of shared dimensions. Related methods have had success in functionally aligning the high-dimensional representations of the human visual cortex along shared latent axes (*38*, *39*). Fourth, while previous work has revealed similarities between the visual cortex representations of humans and monkeys (*40*), we still know little about the degree to which representational dimensions may be universal or species-specific across mammalian vision. This question could be addressed by applying our approach to recordings of cortical responses to the same stimuli in different species.

In sum, our results show that the most brain-aligned representations of visual neural networks are universal and independent of a network’s specific characteristics. What fundamental principles might explain the convergence of networks to universal dimensions? Theories of efficient coding suggest that frequency- and orientation-tuned filters are consistently observed in the first layer of vision systems because they constitute efficient bases that are adapted to the statistics of natural images (*41*, *42*). It remains an open question whether this efficient coding hypothesis can be extended to a deep hierarchy, which could potentially explain universal dimensions as a consequence of optimal image encoding. An alternative possibility is that DNNs learn shared representations of the true generative factors in the visual world—e.g., the objects, materials, contexts, and optical phenomena that make a scene. This could be the case if the optimal strategy for solving challenging tasks on natural stimuli is to learn the invariant properties of reality (*11*), and it would suggest that the universal dimensions detected here reflect a shared internal model of the visual environment in machines and humans.

## MATERIALS AND METHODS

### Natural Scenes Dataset

#### Stimuli and experimental design

The NSD is a large-scale publicly available fMRI dataset on human vision that is described in detail in a previous report (*13*). Here, we briefly review the key attributes of this dataset. The NSD study sourced color natural scene stimuli from the Microsoft COCO database (*12*) and collected 7-T fMRI responses (1.8-mm voxels, 1.6-s repetition time) from eight adult subjects who viewed these stimuli while performing a continuous recognition memory task, namely, to respond if the presented image was seen before in the experiment. Each subject viewed approximately 10,000 stimuli with three repetitions, although some subjects saw fewer stimuli because they did not complete all scanning sessions. Among the stimuli, 872 “shared” images were viewed by all subjects at least once. We used these shared images and the corresponding fMRI data for our main analyses.

#### Data preprocessing

We used the NSD single-trial betas, preprocessed in a 1.8-mm volume space and denoised with the GLMdenoise technique (version 3; betas_fithrf_GLMdenoiseRR). Subsequently, the betas were transformed to *z* scores within each individual scanning session, as recommended by Allen *et al.* (*13*). For all analyses, we used the averaged betas across repetitions.

#### Regions of interest

Our main analyses focused on the nsdgeneral ROI, which was manually drawn and defined on fsaverage by Allen *et al.* (*13*). This ROI contains all voxels in the posterior part of the cortex that were reliably modulated by the presentation of visual stimuli in the NSD experiment, comprising approximately 15,000 voxels in each subject. We also conducted follow-up analyses in smaller ROIs, including the ventral, parietal, and lateral streams, using the “streams” ROIs provided in the NSD dataset.

### Deep neural networks

#### Feature extraction

Before computing our universality and brain similarity metrics, we first needed to extract a set of feature activations from each model layer. We sought to focus on the diversity of encoded image features across different models rather than their variance in representations across space. We thus applied global max pooling to remove spatial information from the activations of each model layer. For convolutional networks, pooling was applied across the height and width dimensions, and for networks with patch embeddings, pooling was applied across patch dimensions. To extract all orthogonal dimensions from each model layer, we first performed PCA on the activations to the 72,128 “unshared” images from NSD, retaining all PCs up to the matrix rank, computed with the default procedure in torch.linalg.matrix_rank in PyTorch (*43*). We then transformed the 872 shared images from NSD to the PC basis and computed universality and brain similarity metrics for each PC. Pooling was applied to both the target features and the predictors. The PCA transformation was only applied to the target features.

#### Model sets

We examined four sets of DNN models in the main results to probe the universality of representational features across different factors of variation: (i) random seeds in trained models, (ii) architectures, (iii) task objectives, and (iv) random seeds in untrained models. The first set included 20 pretrained ResNet-18 models examined in a previous study of model hyperparameters (*15*). These models were initialized with unique random seeds and trained on Tiny ImageNet (*16*). We extracted features from nine rectified linear unit (ReLU) layers that span the depth of the ResNet-18 architecture, except for the final output layer. In total, we extracted 36,596 dimensions from this set.

The second set included 19 pretrained models with varied architectures, which included various convolutional networks, transformers, and MLP-Mixers (table S1). All were trained on ImageNet for object classification (*44*) and obtained from the torchvision library (*43*, *45*). Given the variety of architectures in this set, the sampled layers included ReLU, normalization, attention, multilayer perceptron, and other model-specific operations. The sampled layers span the full range of layer depth in each model (additional details are provided in table S1). The complete list of these layers is included in the Supplementary Materials (*46*). A total of 149,743 dimensions were extracted from this set.

The third set included nine pretrained ResNet-50 models from the torchvision library (*43*) and the VISSL Model Zoo (*47*). These models were trained to perform a variety of tasks on ImageNet images (table S2) (*44*). A total of 43,132 dimensions were extracted from all ReLU layers, except for the final output layer.

The fourth set included 20 untrained ResNet-18 models (*14*) with different random weights, which were created using Kaiming normal initialization (*48*). Each model had a unique random seed. A total of 9413 dimensions were extracted from all ReLU layers. The lower number of dimensions here relative to the set of trained models with different random seeds is due to the low-rank activation matrices of untrained networks.

We also performed a supplementary analysis in a fifth set that included four instance-prototype contrastive learning models trained with varied visual diets (table S3) (*35*). These models used a modified AlexNet architecture (*49*) with group initialization instead of batch normalization layers. A total of 2758 dimensions were extracted from all ReLU layers, except for the final output layer.

### Metrics

#### Universality

Our universality metric estimates the degree to which a representational dimension is shared across multiple DNNs. For a given dimension in a target network, we used cross-validated ridge regression to predict its activations as a linear combination of the activations from another predictor network. We performed this analysis using the same shared NSD images that were used to compute brain similarity (described in the “Brain similarity” section). The regressors consisted of activations concatenated across all sampled layers of the predictor network. The procedure for cross-validated ridge regression is described in the later section. We computed the mean Pearson correlation between the predicted and actual responses of the target dimension across all cross-validation folds, and we repeated this process, using every network other than the target as the predictor. We then obtained the universality score by taking the median correlation across all predictor networks. We used median instead of mean to ensure that the final summary statistic was not driven by a small subset of predictor networks with exceptionally high or low scores. This entire procedure was repeated to obtain universality scores for all dimensions in all sampled layers of the target network, and it was then repeated with each network as the target.

#### Brain similarity

Our brain similarity metric estimates the degree to which a dimension in a DNN can be predicted from fMRI responses measured in the human visual cortex. Given a target dimension from a network, we used cross-validated ridge regression to predict its activations as a linear combination of the trial-averaged fMRI responses to the shared images in a single subject. We computed the mean Pearson correlation between the predicted and actual responses of the target dimension across all cross-validation folds, and we repeated this procedure for all fMRI subjects. Brain similarity is defined as the mean score across all subjects.

#### Permutation tests

We quantified the relationship between universality and brain similarity using nonparametric Spearman correlations, and we obtained *P* values through a permutation test. In this permutation test, we randomly shuffled the image labels before computing universality scores. On each iteration, we then computed a correlation between these permutation-test universality scores and brain similarity. We applied the same shuffling indices to all features within a network to maintain dependencies among network features, and we repeated this procedure 5000 times to obtain a null distribution.

#### Cross-validated ridge regression

We computed universality and brain similarity scores using ridge regression with a nested cross-validation design. The outer loop of this cross-validation design had five folds. For each combination of four folds as training data, we conducted an inner loop of leave-one-out cross-validation to find the optimal ridge penalty for each target feature among zero and values with equal logarithmic spacing between 10^−3^ and 10^4^. We then fit the regression weights using the full set of training data and the optimal ridge penalty, and we applied these regression weights to generate predicted responses in the held-out fold of test data. Performance was evaluated as the correlation between the predicted and actual responses on the held-out test data. This procedure was repeated using all five folds as the held-out test data, and the performance scores were averaged across folds.

#### Alternative mapping methods

We compared two other mapping methods with the ridge regression used for the main results, both conducted with the same fivefold cross-validation design. The first method was ordinary least squares regression without any regularization. The second method is one-to-one mapping: In the training folds, we identified the column in the predictors with the highest Pearson correlation with the target feature and then computed the Pearson correlation between the selected predictor column and the target feature in the test fold.

#### Representational similarity analysis

We computed conventional RSA scores for comparisons of networks and the visual cortex (*22*), adapting the procedure described in (*3*). We split the 872 shared images into a training set and a test set of 436 images each. In each set, RDMs were created by calculating Pearson correlation distances for pairwise comparisons of image representations within each network layer and each fMRI subject. For each network layer, we computed RDMs using the same globally pooled channel activations that were used to compute universality and brain similarity scores. Representational similarities were obtained by calculating the Spearman correlation between the RDMs for a network layer and an fMRI subject, and these scores were averaged across subjects. For each network, the best-performing layer was selected on the basis of the representational similarities in the training set, and the final representational similarity was computed for each network using the selected layer in the held-out test set. We next examined the contribution of universal dimensions to the representational similarities by reducing each network to the subspace spanned by its top 10 or 5 universal dimensions. Specifically, we reconstructed the test-set activations of each network using only the top 10 or 5 most universal dimensions, and we recomputed the final representational similarity on these reconstructed test data.
