# Supplementary material for: Universal dimensions of visual representation
Source: Sci Adv. 2025 Jul 2;11(27):eadw7697. doi: 10.1126/sciadv.adw7697 (PMC12219468; doi:10.1126/sciadv.adw7697)
Supplement: Supplementary file 1 — Figs. S1 to S13 Tables S1 to S3 [file sciadv.adw7697_sm.pdf]

Science Advances
AAAS

Supplementary Materials for

Universal dimensions of visual representation

Zirui Chen and Michael F. Bonner

Corresponding author: Zirui Chen, zchen160@jh.edu

Sci. Adv. **11**, eadw7697 (2025)
DOI: 10.1126/sciadv.adw7697

This PDF file includes:

Figs. S1 to S13
Tables S1 to S3

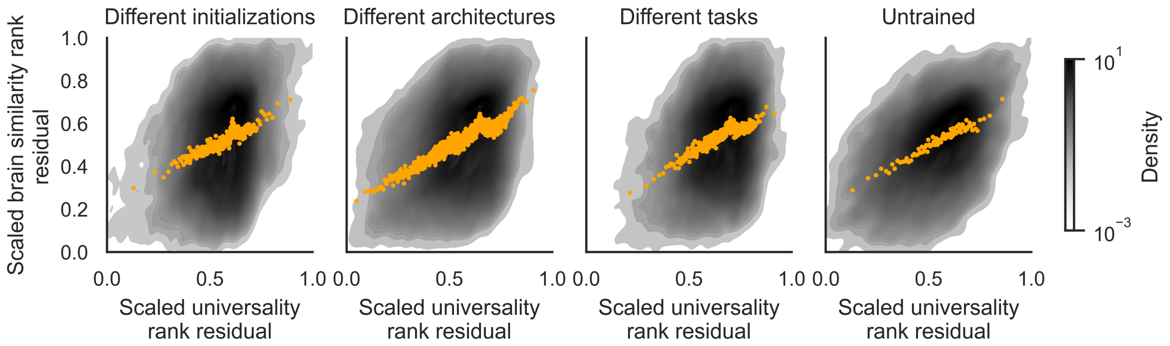

Figure S1: **Residual of universality and brain similarity rank.** These plots show the residuals of universality and brain similarity ranks controlling for the principal component (PC) ranks of the features. Four sets of deep neural networks were examined, including three sets of trained networks with varied initializations, architectures, and tasks and one set of untrained networks. We regressed the rank of the two metrics on PC rank to obtain the residuals, as our partial correlation analysis used Spearman correlation. We further normalized the residuals within each model set to the range $[0, 1]$ by applying min-max scaling. Average residuals of universality and brain similarity rank were computed for equally sized quantiles of 100 dimensions along the x-axis for each network. As reported in the Results, these plots show that the positive association between universality and brain similarity remains significant when controlling for PC rank.

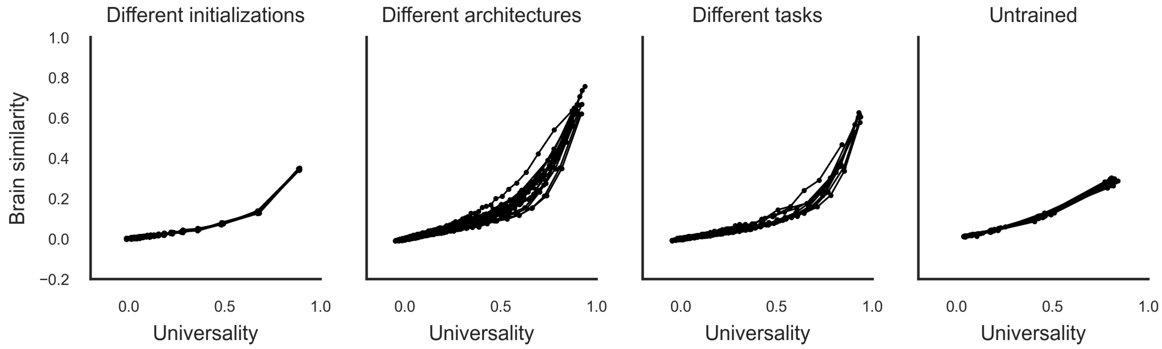

Figure S2: **Universality and brain similarity for individual networks.** These plots show the universality and brain similarity scores for individual networks. The analyses are the same as in Figure 2, but here the results are plotted as the average values for individual networks. Average universality and brain similarity scores were computed for equally sized quantiles of 100 dimensions along the x-axis for each network. As in Figure 2, these plots show that a highly consistent trend is observed across all networks and that universal dimensions are not restricted to a subset of networks (mean Spearman's $\rho = 0.70$, $SD = 0.10$, all $p < 0.0001$; mean partial Spearman's $\rho = 0.39$, $SD = 0.09$, all $p < 0.0001$).

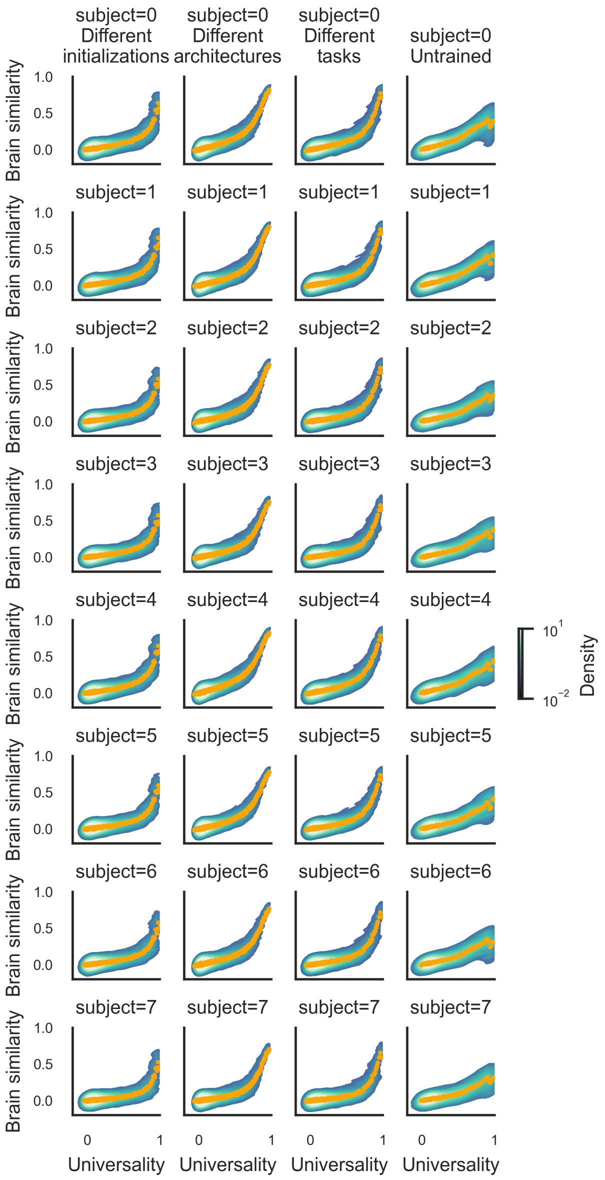

Figure S3: **Universality and brain similarity of network dimensions in individual subjects.** These plots show the relationship between universality and brain similarity in each subject from the fMRI dataset. The analyses are the same in Figure 2 but without averaging the brain similarity scores across subjects. As in Figure 2, these plots show the density of dimensions on a logarithmic scale computed using kernel density estimation. The orange dots show the mean universality and brain similarity scores for equally sized quantiles of 100 dimensions along the x-axis. These results demonstrate that the relationship between universality and brain similarity is highly consistent and robustly detected in all individual subjects (mean Spearman's $\rho = 0.57$, $SD = 0.12$, all $p < 0.0001$; mean partial Spearman's $\rho = 0.26$, $SD = 0.08$, all $p < 0.0001$).

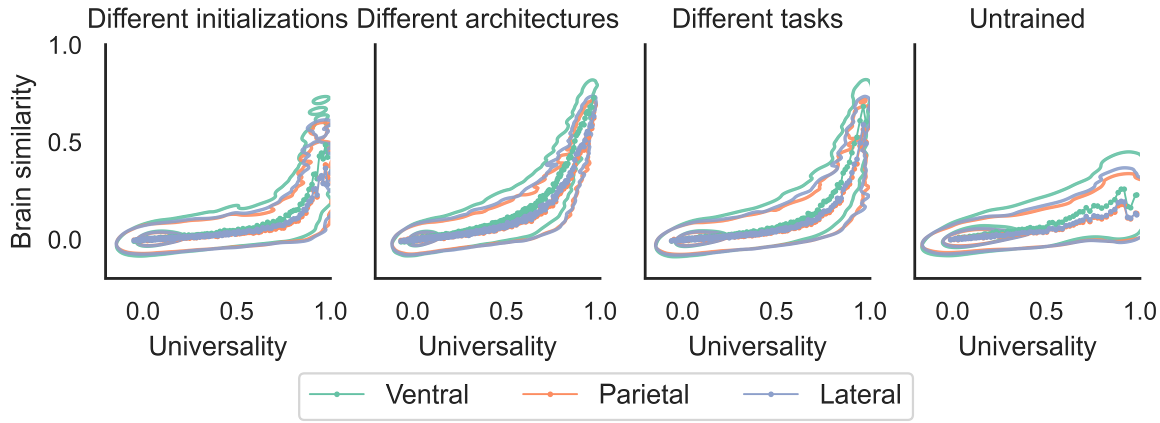

Figure S4: **Universality and brain similarity for multiple regions of interest in visual cortex.** These plots show the universality and brain similarity scores for three regions of interest: the ventral, parietal, and lateral streams. These regions are based on the "streams" masks as defined by the authors of the Natural Scenes Dataset study (*13*). The analysis is the same as in Figure 2, but here brain similarity scores are computed using the three stream regions rather than the large `nsdgeneral` region from our main analyses. To visualize the results for all three regions in a single plot, the contours of the kernel density estimate plots are displayed here, rather than their density values. Average universality and brain similarity scores were computed for equally sized quantiles of 100 dimensions along the x-axis for each region. These plots show that a highly consistent trend is observed across all regions (mean Spearman's $\rho = 0.58$, $SD = 0.10$, all $p < 0.0001$; mean partial Spearman's $\rho = 0.26$, $SD = 0.08$, all $p < 0.0001$).

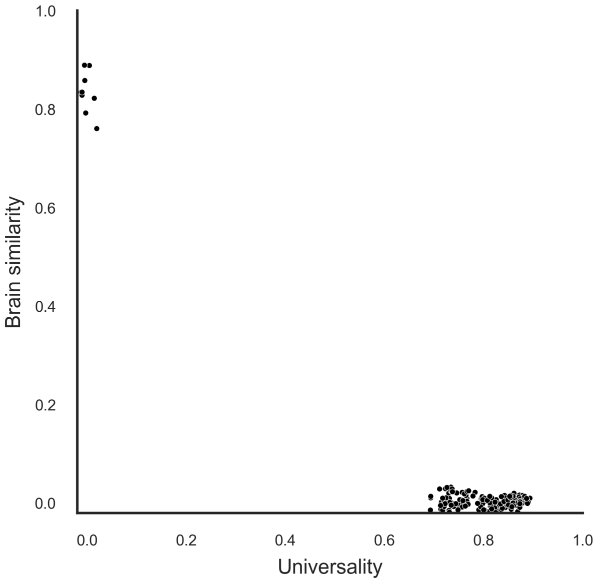

Figure S5: **Universality and brain similarity are not intrinsically correlated.** Universality and brain similarity scores were computed on simulated data to demonstrate that these metrics are not intrinsically correlated and can be trivially dissociated from one another. Data were generated for 20 simulated "subjects" and "networks." All subjects and a single network were created from a common matrix of orthonormal random variables with added Gaussian noise. The remaining networks were created from another matrix of orthonormal random variables with added Gaussian noise. These simulated data yield a subset of dimensions with low universality and high brain similarity and another set of dimensions with high universality and low brain similarity. The plot limits for both axes are set to [-0.02, 1] to make the data points near 0 visible.

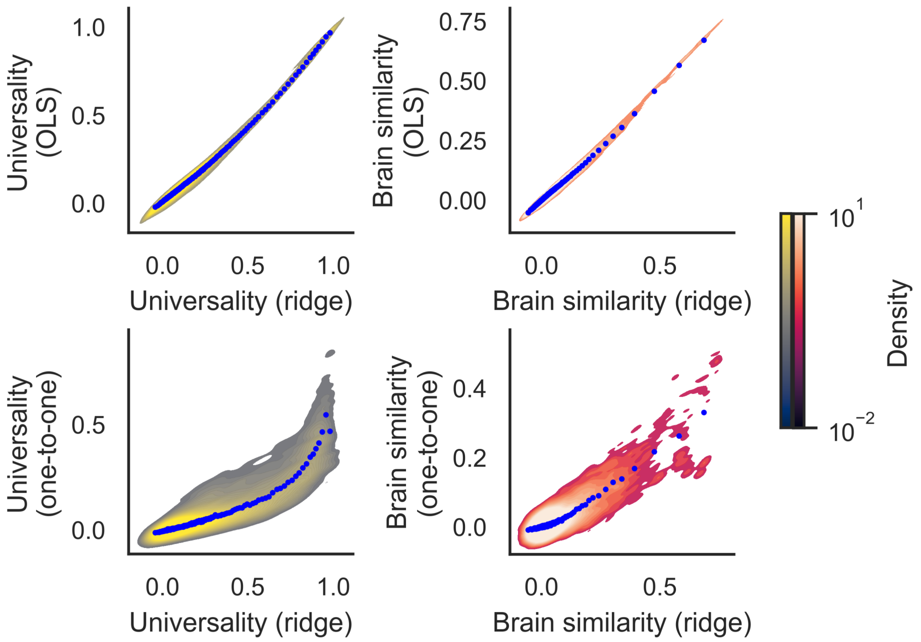

Figure S6: **Universality and brain similarity computed with different mapping methods** Universality and brain similarity were computed for representational dimensions in the set of 20 ResNet-18 architectures trained on image classification using the Tiny ImageNet dataset (*14–16*), initialized with varied seeds. Plots in each row compare the metrics computed with the default ridge regression method to those computed with ordinary least square (OLS) regression or one-to-one mapping. These plots show the default metrics on the x-axis and the alternative metrics on the y-axis, with the density of dimensions computed using kernel density estimation. The blue dots show the mean universality and brain similarity scores for equally sized quantiles of 200 dimensions along the x-axis. The results show that these metrics are not strongly contingent on the use of regularized regression and that similar trends are observed even without regression-based reweighting (i.e., with one-to-one mapping). Note that, as expected, the use of one-to-one mapping makes all the values lower, but the resulting values are nonetheless strongly correlated with those obtained with the ridge regression procedure.

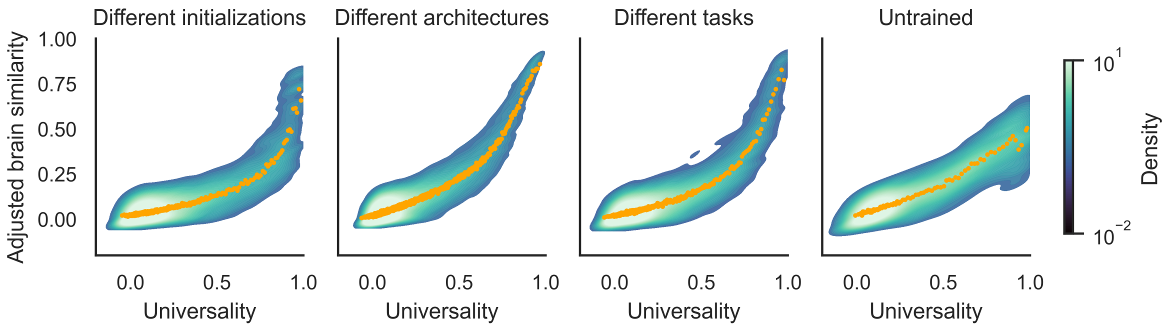

Figure S7: **Universality and adjusted brain similarity of network dimensions** These plots show the relationship between universality and brain similarity adjusted by the inter-subject reliability of the fMRI data. Because brain similarity is calculated as a correlation between a network dimension and a linear projection of the fMRI data, we can compute the relevant fMRI reliability as the average between-subject correlation of the projected fMRI responses. These between-subject correlations were calculated on each test fold and averaged across folds. Noise-ceiling adjusted brain similarity scores were computed by dividing the original brain similarity by the square root of the between-subject reliability (with negative values set to zero). Four sets of deep neural networks were examined, including three sets of trained networks with varied initializations, architectures, and tasks and one set of untrained networks. As in Figure 2, these plots show the density of dimensions on a logarithmic scale computed using kernel density estimation. The orange dots show the mean universality and adjusted brain similarity scores for equally sized quantiles of 100 dimensions along the x-axis. The results are highly similar to those shown in Figure 2 using unadjusted brain similarity scores (mean Spearman's $\rho = 0.69$, $SD = 0.09$, all $p < 0.0001$; mean partial Spearman's $\rho = 0.37$, $SD = 0.08$, all $p < 0.0001$).

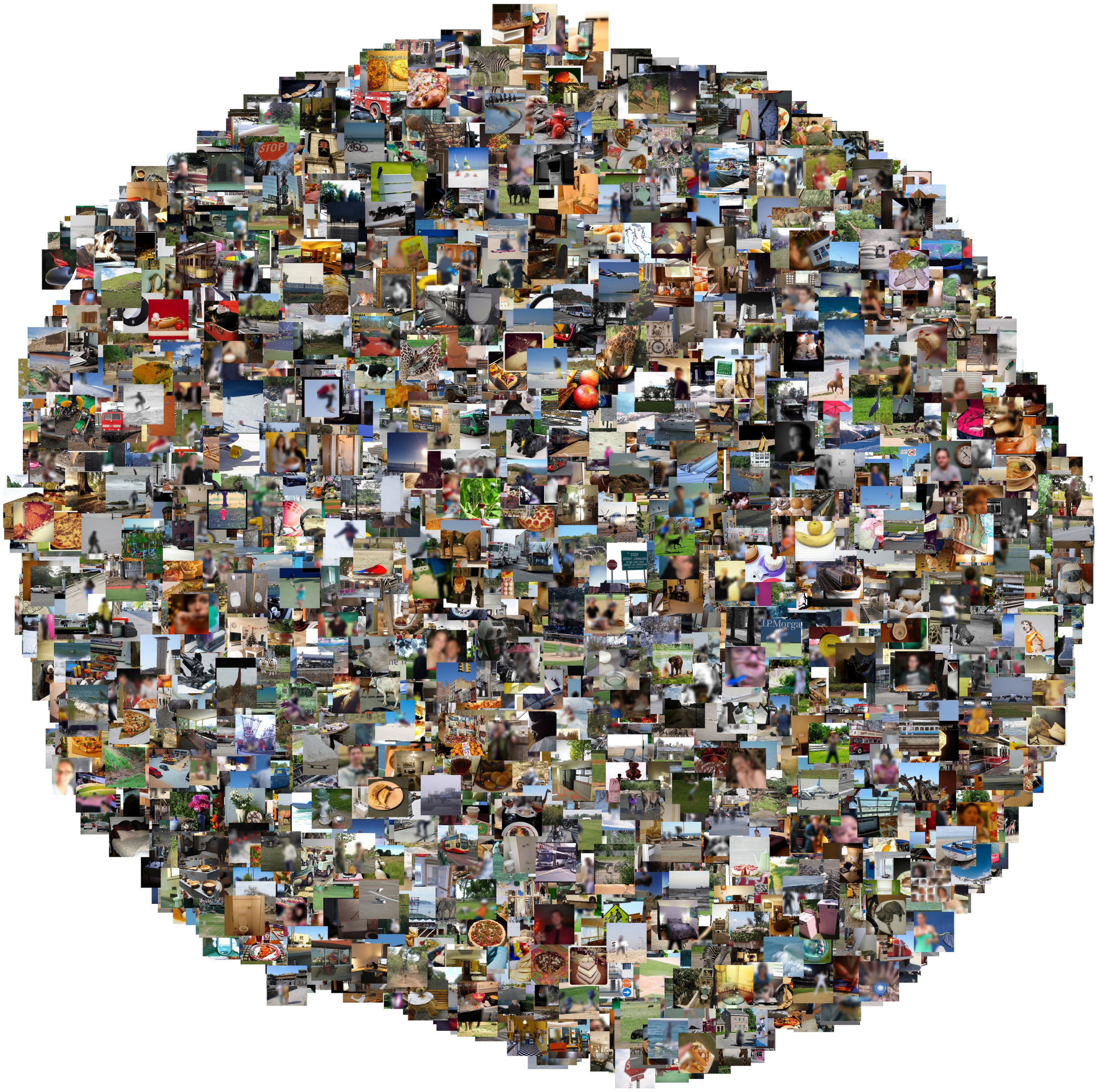

Figure S8: **Two-dimensional visualization of model-specific representations.** Image activations for the 100 *least* universal dimensions from a high-level network layer were embedded in two dimensions using uniform manifold approximation and projection. Specifically, image activations were obtained for the 100 dimensions with the lowest universality scores in the penultimate layer from the set of ResNet-50 models trained on different tasks. In contrast to the universal dimensions visualized in Figure 4, this plot shows no clear semantic organization.

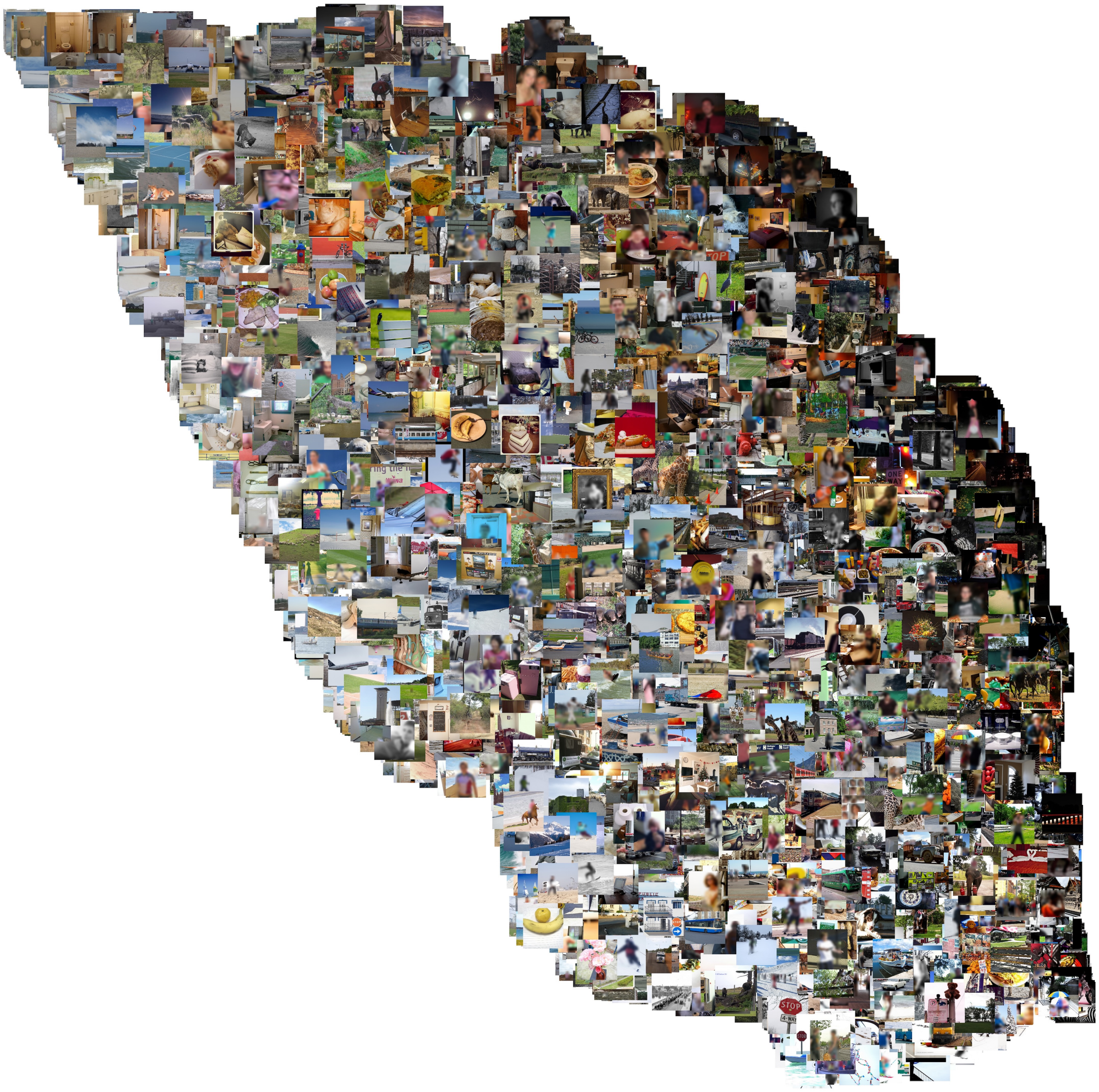

Figure S9: **Two-dimensional visualization of untrained models.** Image activations for the 100 most universal dimensions of untrained networks were embedded in two dimensions using uniform manifold approximation and projection. Specifically, image activations were obtained for the top 100 dimensions with the highest universality scores in the penultimate layer from the set of untrained ResNet-18 models with different random weights. In contrast to the universal dimensions visualized in Figure 4, this plot shows no clear semantic organization. Instead, the universal dimensions of untrained networks appear to emphasize low-level image properties, as demonstrated by the strong luminance gradient from left to right in this plot.

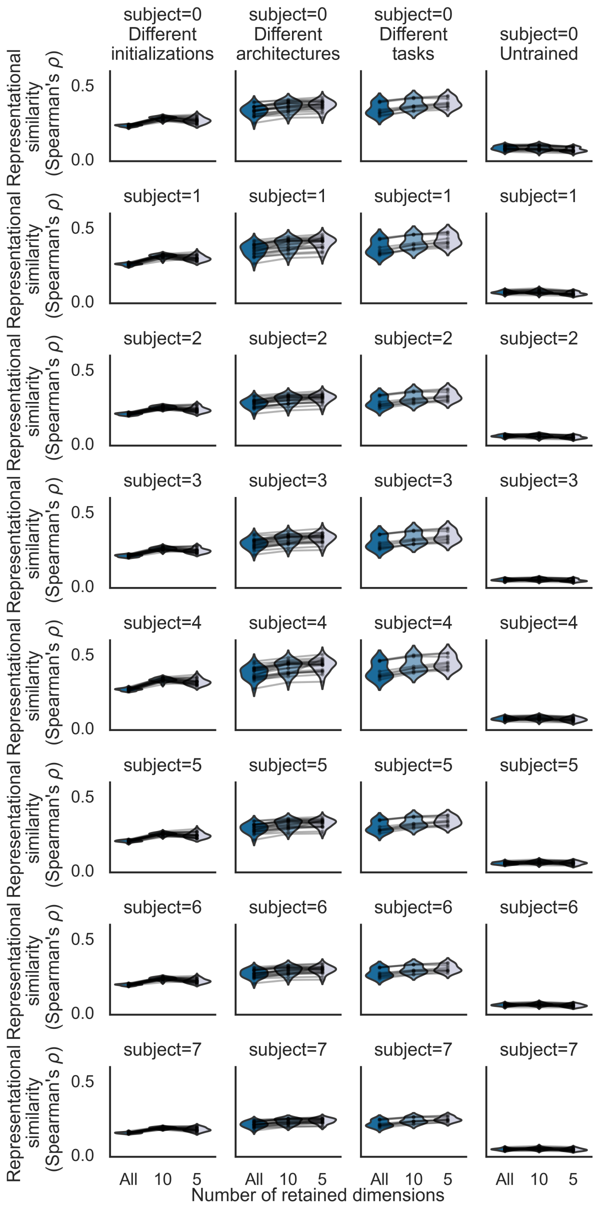

Figure S10: **Universal dimensions underlie the results of representational similarity analyses for individual subjects.** These plots show the results of representational similarity analyses (RSA) comparing networks with each subject from the fMRI dataset. The analyses are the same as in Figure 5 but without averaging the representational similarities (Spearman's ρ) across subjects. As in Figure 5, each dot is a network, whose representations were either intact or reduced to subspaces of their top ten or five universal dimensions, and the violin plots show distributions of representational similarities across networks. These results demonstrate that the subspaces of universal dimensions within each network consistently drive the representational similarity between neural networks and visual cortex across all individual subjects.

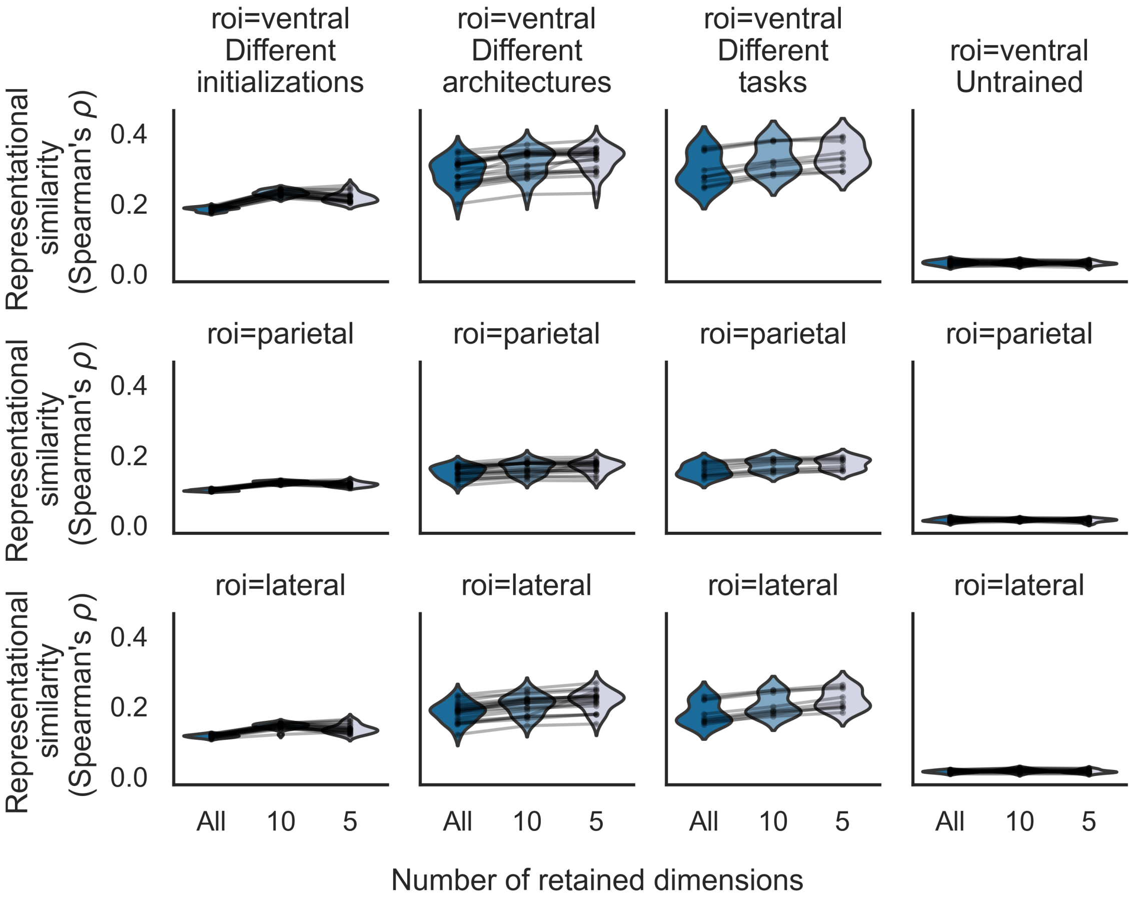

Figure S11: **Universal dimensions underlie the results of representational similarity analyses in multiple regions of interest.** These plots show the results of representational similarity analyses (RSA) comparing networks with the fMRI responses from three regions of interest: the ventral, parietal , and lateral streams. These regions are based on the "streams" masks as defined by the authors of the Natural Scenes Dataset study (*13*). The analyses are the same as in Figure 5, but here the representational dissimilarity matrices (RDM) for the fMRI data are computed using the three stream regions rather than the large nsdgeneral region from our main analyses. As in Figure 5, each dot is a network, whose representations were either intact or reduced to subspaces of their top ten or five universal dimensions, and the violin plots show distributions of representational similarities (Spearman's ρ) across networks. These results demonstrate that the subspaces of universal dimensions within each network consistently drive the representational similarity between neural networks and visual cortex across all regions.

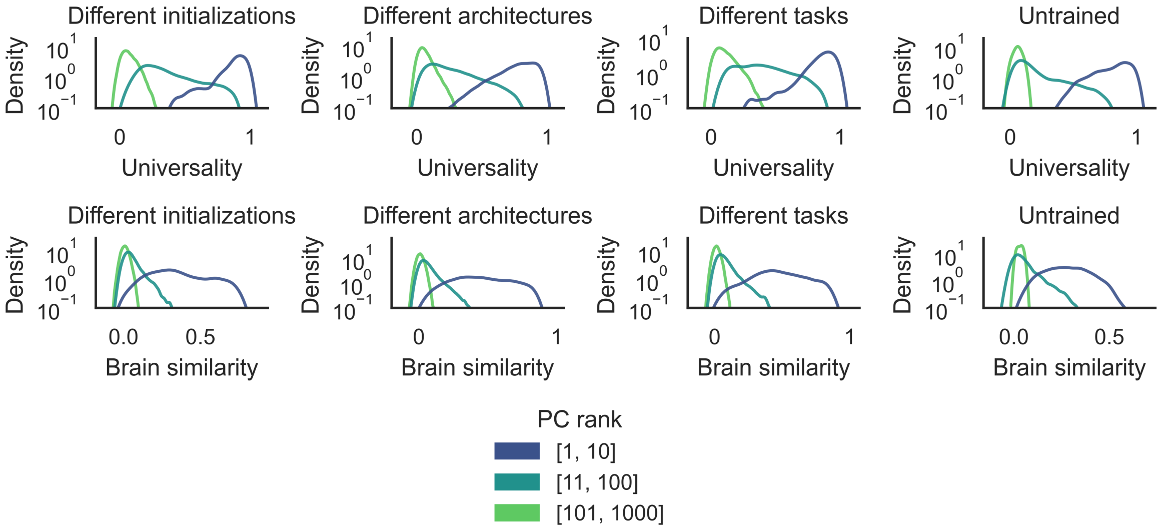

Figure S12: **Distributions of universality and brain similarity for three decades of PC ranks** These plots show distributions of universality and brain similarity scores for PCs extracted from each model layer in the four sets of networks shown in Figure 2. These distributions are plotted for three different decades of PC ranks using kernel density estimation. While there is a general trend for lower-rank PCs to have higher universality and brain similarity scores, there is nonetheless wide variation within each decade of PC ranks, with the scores from the first decade of ranks ranging as low as those from the third decade.

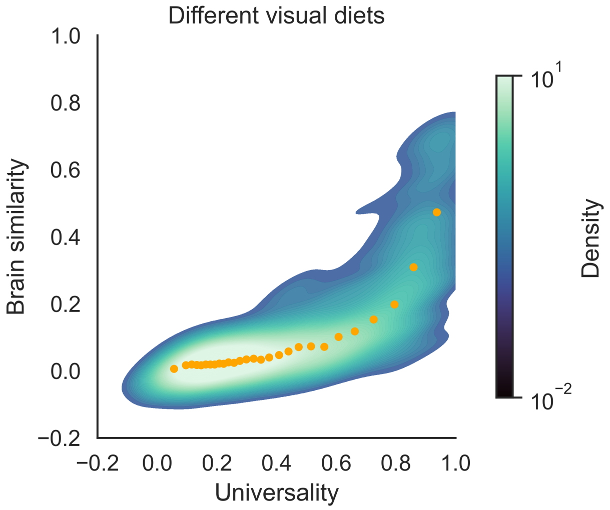

Figure S13: **Universality and brain similarity of neural networks trained with varied visual diets** This plot shows the relationship between universality and brain similarity of representational dimensions extracted from a set of models trained with varied visual diets but matched on architecture and task objective. The analysis is the same as in Figure 2, and the plot shows the density of dimensions on a logarithmic scale computed using kernel density estimation. The orange dots show the mean universality and brain similarity scores for equally sized quantiles of 100 dimensions along the x-axis. As in Figure 2, this plot exhibits a high density of points near the origin, showing that most dimensions are idiosyncratic to each network and are not shared with the human brain. However, there is also a subset of dimensions with exceptionally high universality and brain similarity scores. These latter dimensions correspond to representations that are consistently learned by networks with varied visual diets and that are also strongly shared with the visual representations of the human brain.

Table S1: **Networks from the set of trained models with varied architectures.**

Architecture	Learning objective	Architecture type	Training data	Source
ResNet18	Object classification	Convolutional	ImageNet	PyTorch
ResNet50	Object classification	Convolutional	ImageNet	PyTorch
ResNeXT50_32x4d	Object classification	Convolutional	ImageNet	PyTorch
Wide_ResNet50_2	Object classification	Convolutional	ImageNet	PyTorch
AlexNet	Object classification	Convolutional	ImageNet	PyTorch
VGG16	Object classification	Convolutional	ImageNet	PyTorch
DenseNet121	Object classification	Convolutional	ImageNet	PyTorch
SqueezeNet1_1	Object classification	Convolutional	ImageNet	PyTorch
ShuffleNet_v2_x1_0	Object classification	Convolutional	ImageNet	PyTorch
ConveNeXt_tiny	Object classification	Convolutional	ImageNet	PyTorch
Swin_t	Object classification	Transformer	ImageNet	PyTorch
MaxVit_t	Object classification	Transformer	ImageNet	PyTorch
Cait_xxs24_224	Object classification	Transformer	ImageNet	Timm
Coat_lite_tiny	Object classification	Transformer	ImageNet	Timm
Deit_tiny_patch16_224	Object classification	Transformer	ImageNet	Timm
Levit_128	Object classification	Transformer	ImageNet	Timm
Mixer_b16_224	Object classification	MLP-Mixer	ImageNet	Timm
ResMLP_12_224	Object classification	MLP-Mixer	ImageNet	Timm
Dla34	Object classification	Convolutional	ImageNet	Timm

Table S2: Networks from the set of trained models with varied training objectives.

Architecture	Learning objective	Training setting	Training data	Source
ResNet50	Object classification	Supervised	ImageNet	PyTorch
ResNet50	Jigsaw	Self-supervised	ImageNet	VISSL
ResNet50	RotNet	Self-supervised	ImageNet	VISSL
ResNet50	ClusterFit	Self-supervised	ImageNet	VISSL
ResNet50	NPID++	Self-supervised	ImageNet	VISSL
ResNet50	PIRL	Self-supervised	ImageNet	VISSL
ResNet50	SimCLR	Self-supervised	ImageNet	VISSL
ResNet50	SwAV	Self-supervised	ImageNet	VISSL
ResNet50	DeepClusterV2	Self-supervised	ImageNet	VISSL

Table S3: Networks from the set of trained models with varied visual diets.

Architecture	Learning objective	Training data	Source
AlexNet-GN	IPCL	ImageNet	IPCL
AlexNet-GN	IPCL	OpenImages	IPCL
AlexNet-GN	IPCL	Places2	IPCL
AlexNet-GN	IPCL	VGGFace2	IPCL
